# Legacy of Polio—Use of India’s Social Mobilization Network for Strengthening of the Universal Immunization Program in India

**DOI:** 10.1093/infdis/jix068

**Published:** 2017-07-01

**Authors:** Nicole Deutsch, Prem Singh, Vivek Singh, Rod Curtis, Anisur Rahman Siddique

**Affiliations:** 1 Polio Section, UNICEF India, New Delhi, and; 2 Indian Institute of Public Health, Public Health Foundation of India, Hyderabad, Telangana;; 3 Polio Section, UNICEF Regional Office of South Asia, Kathmandu, Nepal

**Keywords:** Social Mobilization Network (SMNet), Polio Legacy, System Strengthening, Universal Immunization Program (UIP).

## Abstract

The Social Mobilization Network (SMNet) has been lauded as one of the most successsful community engagement strategies in public health for its role in polio elimination in India. The UNICEF-managed SMNet was created as a strategy to eradicate polio by engaging >7000 frontline social mobilizers to advocate for vaccination in some of the most underserved, marginalized, and at-risk communities in India. This network focused initially on generating demand for polio vaccination but later expanded its messaging to promote routine immunization and other health and sanitation interventions related to maternal and children’s health. As an impact of the network’s interventions, in collaboration with other eradication efforts, these high-risk pockets witnessed an increase in full routine immunization coverage. The experience of the SMNet offers lessons for health-system strengthening for social mobilization and promoting positive health behaviors for other priority health programs like the Universal Immunization Program.

Polio eradication efforts in India and globally have demonstrated that, in many locations, the critical path to success lies in complementing a focus on biological issues with a comprehensive address of the sociocultural challenges [1]. Experience has shown that the most tenacious pockets of wild poliovirus circulation are entrenched in either hard-to-reach or underserved communities with deep-rooted sociocultural resistance to immunization. Polio eradication in these contexts in India became possible because of the support of a social mobilization and behavior change communication strategy, which encompassed a range of equity-focused community engagement efforts to overcome these challenges in the most at-risk communities [2–7].

India’s last polio case occurred on 13 January 2011, and the country was certified polio free in 2014, after many years of concerted efforts led by the government of India with ardent support from partners, including the World Health Organization (WHO), UNICEF, and Rotary. In an effort to eradicate polio, the world’s largest coordinated vaccination campaigns were launched, with millions of frontline workers vaccinating 170 million children aged <5 years in each national polio round [8, 9]. A major contributor to the vaccinators’ success in highest-risk polio-priority areas was the Social Mobilization Network (SMNet), a 4-tiered community mobilization and supervisory structure with, at its base, a cadre of >6000 Community Mobilization Coordinators (CMCs), of which >90% were women from the local communities. (Community Mobilization Coordinators are key cadres for social mobilization activities at the community level and constitute >90% [95% in Bihar, 98% in Uttar Pradesh, and 99% in West Bengal] of the SMNet human resources.) This network, managed by UNICEF, was created in the polio-endemic and most operationally challenging states of Uttar Pradesh and Bihar and expanded in 2011 through a network of nongovernmental organizations in the state of West Bengal as an emergency response to what would be India’s last wild poliovirus case—in Howrah district, close to Kolkata [10].

The SMNet originated due to a need to interpret and engage the role of social determinants in the transmission and outbreak of wild poliovirus in these states. At the time, Uttar Pradesh—India’s most populous state, with poor sanitation and hygiene, a high enteric disease burden, high population density, and a large birth cohort—offered favorable conditions for poliovirus to spread and, as a result, regularly recorded the highest volume of polio cases in the world [11]. Vaccination coverage in polio campaigns was insufficient to stop transmission, particularly in localized Muslim communities where rumors about the vaccine led to refusal rates of up to 20% [12–14]. As a result, the disease burden among the Muslim community was over-represented, with about 59% of cases in Uttar Pradesh occurring in Muslim children, despite constituting only 12% of the population. Data showed that a Muslim child was 5 times more likely not to receive even 1 dose of oral polio vaccine [15, 16]. Similarly, Bihar was afflicted by higher rates of refusal to accept the polio vaccine, especially in minority and underserved communities. Both states also experienced ongoing polio transmission in their large mobile and migrant communities and in remote, difficult-to-access areas with often underserved populations [17].

The SMNet was tasked with identifying and engaging with these high-risk groups in high-risk areas to ensure all children aged <5 years were vaccinated against polio in every round. As part of the 107 Block Plan, the network introduced convergent messaging promoting routine immunization, diarrhea prevention and management, handwashing with soap, and exclusive breastfeeding practices as a means to both tackle underlying causes for ongoing polio transmission and alleviate resistance by holistically addressing concerns of underserved populations.(Full routine immunization coverage is the percentage of 1-year-old children in SMNet areas who received 1 dose of BCG vaccine, 3 doses of diphtheria- pertussis-tetanus vaccine, 3 doses of oral polio vaccine, and 1 dose of measles vaccine. These data are generated through field books [name-based reporting of 0–1-year-old children] on a monthly basis and reported through SMNet management information system.)

As part of the Polio Endgame Strategy, the SMNet increasingly focused on boosting full immunization through engaging policy, strengthening capacity development, and monitoring for communication activities, social mobilization, media sensitization, supportive supervision, and evidence-based, real-time communication planning.

## CRITICAL REVIEW OF THE SOCIAL MOBILIZATION NETWORK STRATEGY FOR STRENGTHENING THE UNIVERSAL IMMUNIZATION PROGRAM

**Figure 1.**
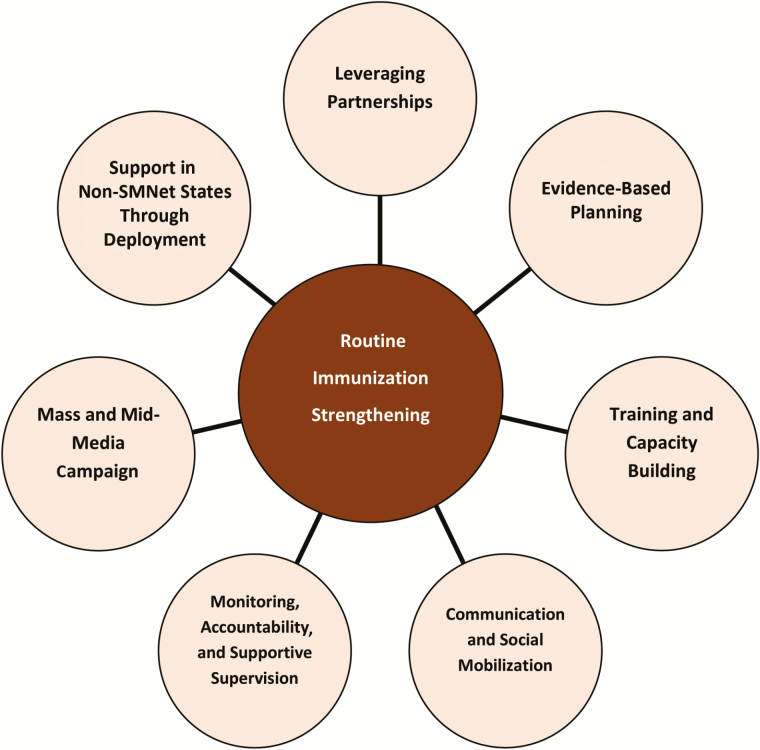
Universal Immunization Program strengthening strategies: lesson learned from polio eradication.

This article describes the key strategies of the SMNet, which were designed to support polio eradication and have since been applied to routine immunization and broader health system strengthening in India. The network developed and demonstrated key strategies such as evidence-based communication planning and microplanning, development and maintenance of interpersonal communication skills for mobilization of individuals and groups, strong outreach and advocacy, systematic building of an effective partnership for communication and strong supervision, and accountability for action (Figure 1). These strategies are significant for the transition of the network into supporting broader health services and can be considered for other programs which depend on community orientation for better uptake of services.

## LEVERAGING EFFECTIVE PARTNERSHIPS

The SMNet’s success was built on a strong polio partnership with multiple stakeholders, such as tgovernment agencies; development partners, including WHO, Rotary, and CORE group and local community-based institutions. (CORE Group emerged organically, in 1997, when a group of health professionals from nongovernmental development organizations saw the value of sharing knowledge and ideas about how to best help children survive. In India, CORE group is working for polio eradication program in few districts of Uttar Pradesh and Bihar.) Government partners included the Department of Women and Child Development, Rural Development Department, National Cadet Corps, Indian Railways, and paramilitary forces. A seamless partnership with the WHO–National Polio Surveillance Program helped in joint microplanning, monitoring and supportive supervision practices, and the development of various training resources for training and capacity building of health functionaries. The Indian Medical Association, India Academy of Pediatrics, and several universities also played important roles in supporting the program.

A core strategy of the program was to engage leaders at all levels, especially local and national levels. Perhaps more important, the local officials (Panchayat Raj Institutions) were involved in mobilizing their communities- building “polio gates,” inaugurating booths/campaigns and ensuring proper visibility of materials. (Panchayat or Panchayati Raj is a system of governance in which gram panchayats [village level] are the basic units of administration. It has 3 levels: village, block, and district. The term *panchayat raj* originated during the British administration. *Raj* literally means governance or government.) District and block officials were responsible for overseeing task forces and evening meetings, which triggered corrective action both daily and strategically to ensure continually improving, high-quality campaigns.

In response to the high rates of polio and resistance in some Muslim areas, the SMNet made concerted efforts to engage religious leaders. In 2004, the SMNet launched the Underserved Strategy to address concerns of socioeconomically underprivileged, minority Muslim communities in western Uttar Pradesh. The crux of the strategy was to involve these communities in the planning, implementation, and monitoring of the polio program, routine immunization, and other convergent health issues in order to gain full ownership and acceptance. Crucially, the SMNet workforce was overwhelmingly female —allowing them access to mothers inside households—and drawn from the precise community in which they worked. Religious leaders were engaged and informed about the polio program and requested to serve as influencers, working with the teams to counter household religious refusals. They also played a crucial role in motivating minority communities to accept the CMCs and information and services provided by them. Faith-based institutions were engaged at the highest levels, and on the ground, more than 16000 mosques were mobilized to regularly make announcements on polio and routine immunization sessions. Religious organizations and academic institutions such as Aligarh Muslim University directly contributed toward acceptance of the strategy by developing pamphlets reflective of the health benefits of the Universal Immunization Program. These institutions organized immunization camps, rallies, and information to improve immunization coverage across Uttar Pradesh and Bihar. As of 2016, 49266 influencers have been identified and engaged by the SMNet to systematically support polio immunization and the Universal Immunization Program. These include religious leaders, doctors and health practitioners, quacks and rural medical practitioners, teachers, government workers, community-based women groups, Panchayat Raj Institution officials, community leaders and elected representatives, shopkeepers, and brick-kiln owners [18].

## HUMAN RESOURCE CAPACITY BUILDING

The SMNet strategy placed strong emphasis on both formal and informal trainings, with all workers regularly undertaking capacity assessments for maintaining high technical competence. Formal training included induction training (at joining), refresher training (3 months after joining), and annual interpersonal communication training. Several forms of informal trainings were conducted, with particular emphasis on supportive supervision visits where the SMNet was working. At all levels, a culture was developed where the identification of areas of programmatic weakness and capacity/knowledge gaps was considered good practice and rewarded. These trainings also contributed toward capacity building and skill development of frontline workers such as Accredited Social Health Activists, Anganwadi Workers, and Auxiliary Nurse Midwives, particularly in Bihar, where these cohorts were systematically engaged ahead of all campaigns. (Details are given in Table 1.) In SMNet states in 2015 alone, 62307 auxiliary nurse midwives, 135411 accredited social health activists, and 68395 Anganwadi workers were trained by the polio program to improve interpersonal communication skills related to the Universal Immunization Program.

**Table 1.** Role of Various Frontline Health Workers

Health personnel	ASHA	ANM	AWW
About	Consists of community health workers instituted by the government of India’s Ministry of Health and Family Welfare as part of the National Rural Health Mission.	Consists of village-level female health workers in India who serve as the first contact between the community and the health services. Regarded as grass-roots workers in the health organization pyramid.	*Anganwadi* means “courtyard shelter” in Indian languages. Started by the Indian government in 1975 as part of the Integrated Child Development Services program to combat child hunger and malnutrition.
Type of compensation	Local volunteers receive outcome-based remuneration and financial compensation	Permanent or contractual salaried health manpower	Salaried manpower under Department of Women and Child Development
Level of education	Minimum eighth standard pass	Auxiliary nurse midwife nursing course	10 + 2
Training status	7 training modules under National Health Mission. ANM acts as a resource for the training of ASHAs.	Nursing course and various health programs trainings	Job training course and refresher trainings through Anganwadi training center
Major job profile	Work as first port of call for health and act as a link between community and health department. Provide health information to community, counsel and mobilize community for health services. Act as a depot holder for key drugs and logistics.	Work at health subcentres. Expected to be multipurpose health workers. Work includes maternal and child health along with family planning services, health and nutrition education, efforts for maintaining environmental sanitation, immunization, control of communicable diseases, treatment of minor injuries, and first aid in emergencies and disasters.	Work includes conducting regular quick surveys of all families, organizing preschool activities, providing health and nutrition education to families, especially pregnant women on how to breastfeed, motivating families to adopt family planning, educating parents about child growth and development, providing supplementary nutrition to children aged <6 years and pregnant women, educating teenage girls and parents by organizing social awareness programs, etc.
Population served	One worker for 1000 population	One worker for 5000 population in plains and 3000 population in hilly and tribal areas	One worker for 1000 population

Abbreviations: ANM, Auxiliary Nurse Midwives; AHSA, Accredited Social Health Activists; AWW, Anganwadi Workers.

## EVIDENCE-BASED PLANNING

Microplanning was a key component of the SMNet’s success at the field level, bringing greater focus and efficiency to planning and executing communication activities. The communication microplans at all levels were based on the need and prepared with comprehensive mapping of households and issues by the CMCs. These microplans evolved into field books containing the names, ages, and vaccination statuses of all children aged <5 within a CMC’s area and facilitated the development of a focused “due list” of beneficiaries for polio and Universal Immunization Program rounds, as well as the easy identification of in- or outgoing mobile and migrant groups. Microplans for the Universal Immunization Program have been continually reviewed and revised by the WHO and UNICEF polio network to ensure completeness of Universal Immunization Program service reach. The SMNet microplans have led to the identification and coverage of almost 256000 migrant sites with 166000 high-risk areas identified as a settled population. Government, WHO, and UNICEF SMNet jointly identified 400000 high-risk areas with nonsettled populations, such as brick kilns, construction sites, nomads, and so on, to be included in Universal Immunization Program microplans to directly improve the coverage of hard-to-reach and underserved populations often living outside the existing health system. Using a network of 50324 “informers” (often nearby shopkeepers, etc), the SMNet systematically tracked >2000 sites with highly mobile and migrant group traffic, covering 83000 families (61000 children aged <5) that were left out in earlier rounds.

## COMMUNICATION AND SOCIAL MOBILIZATION APPROACHES FOR BEHAVIOR CHANGE

The 3-tiered structure of the SMNet galvanizes social mobilization and communication for behavior change at the district, block, and community level via District, Block and Community Mobilization Coordinators in areas where demand and service delivery is poor. The SMNet uses a multipronged communications approach encompassing (1) mass and mid-media approaches (using tested materials developed nationally by UNICEF for the government and partners); (2) information, education, and communication materials; (3) ground-level community engagement strategy engaging community influencers, religious leaders, house-to-house mobilization, and mothers meetings; and (4) the use of key community sites such as mosques, schools, or religious festivals to generate demand.

### Community Mobilization (Interpersonal Communication and Meetings)

The CMCs of the SMNet are overwhelmingly women aged 25–45 years with basic literacy skills and are responsible for 350–500 households. They are drawn from the same community where they work and have the same religious beliefs and language/ dialect. They support the Universal Immunization Program by mobilizing communities through house-to-house interpersonal communication and group counselling sessions addressing myths and misconceptions and ensuring correct knowledge about vaccines. The CMCs mobilize families, hold mothers’ meetings and religious meetings, coordinate mosque announcements, and conduct polio classes in schools, madrasas, and other congregations. Each month, the network holds >7000 mothers’ meetings and >200000 individual interpersonal communication sessions for routine immunization (SMNet Management Information System, unpublished data), reaching out to approximately 2.53 million households to inform parents of the timing and location of upcoming sessions and if and when their children are due for vaccination. On the day of the Universal Immunization Program session, the CMCs are required to be present at the session site, working from a checklist that monitors whether the vaccinator is in attendance; whether all vaccines and other hardware, including needles and sharp boxes, have been supplied; and whether caregivers have been informed about the date of their next required immunization for their child and the risks of adverse events following immunization. These monitoring forms are then referred by the Block and District Mobilization Coordinators to the district authorities for real-time feedback and corrective actions. Community Mobilization Coordinators use the opportunity of the session site to further counsel the community on diarrheal control (use of oral rehydration solution and zinc), exclusive breastfeeding, hand washing, and toilet use/conversion).

### Media Engagement

**Figure 2.**
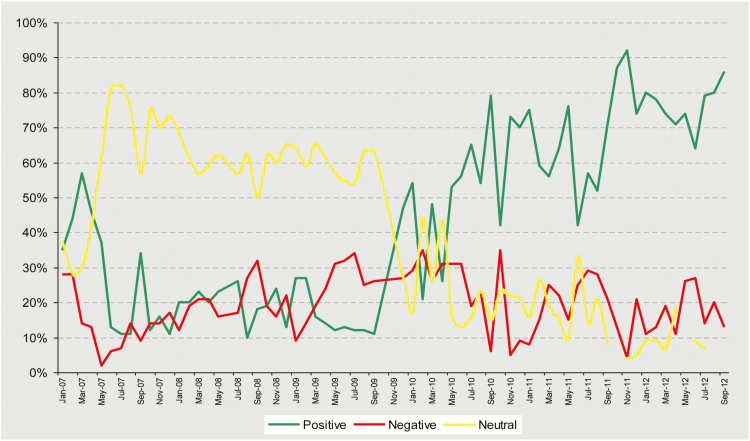
Trend tonality of polio news stories from Western Uttar Pradesh from January 2007 to September 2012. Source: UNICEF [18].

A systematic media engagement strategy was used in high-risk states, supported by human resources capacity at the national and state levels within UNICEF. This strategy used regular media workshops for electronic and print media and facilitated field visits, briefings, and campaign launches to improve the quality and tonality of reporting and to create a supportive media environment, which played a key role in reaching out to high-risk populations. Appeals from political and religious leaders in the print media and the use of local channels to answer localized rumors also helped to build faith in vaccines. (see media tonality graph in Figure 2)

## MONITORING, EVALUATION, AND SUPPORTIVE SUPERVISION

Regular and timely monitoring, which captures data through simple monitoring formats that feed into real-time campaign planning and decision making, was an indispensable factor behind the success of the SMNet. There were 3 forms of monitoring, evaluation, and learning activities under the program: (1) immunization reporting system, (2) concurrent monitoring (both operations and communication) and supportive supervision, and (3) evaluation studies such as Knowledge, Attitude, and Practice (KAP) studies and SMNet evaluations. The main feature of the SMNet monitoring system was the capacity for real-time analysis of collected data and feedback every night during campaigns (for in-course corrective action) and for analysis prior to monthly campaign review and preparation meetings. This enabled the SMNet functionaries to identify issues for targeted response (a cluster of religious refusals would generate a visit from an imam, for instance), as well as to track individual performance and take corrective actions if targets were not being achieved or significant deviations were evident.

Following the “end” of polio in India, UNICEF’s SMNet has been used by the government of India for monitoring of communication activities related to Mission Indradhanush, the immunization campaign. UNICEF helped to develop a new monitoring tool to collect data from >1000 monitors (which included the senior cadres of the SMNet who had been relocated to support poor-performing routine immunization states) to generate a dashboard for the government to take immediate corrective actions during the campaign. This resulted in improvement of communication indicators and coverage during successive Mission Indradhanush campaigns.

**Mission Indradhanush**

A national campaign to boost routine immunization in poor-performing states and districts was launched in 2015 and used some strategies developed by the polio program to boost full immunization, particularly in high-risk areas and groups. More than 500 SMNet members were deployed from Bihar and Uttar Pradesh across 52 districts of non-SMNet high-priority states like Madhya Pradesh, Rajasthan, Haryana, and Chhattisgarh to augment the communication activities for demand generation in these states, supporting communication planning, implementation, advocacy, social mobilization, monitoring, and supportive supervision. More than 200 additional members were deployed within the SMNet states of Uttar Pradesh and Bihar to non-SMNet areas to support communication activities under Mission Indradhanush, focused on coaching local officials and frontline workers on strategies to effectively campaign to reach every child. Through SMNet support and effective monitoring of communication activities, district communication plans were updated in 94% of deployed districts (Madhya Pradesh: 97%, Rajasthan: 75%, Haryana: 100%, Chhatisgarh: 100%; compared with 75% nationally) (Communication Monitoring Data, unpublished data).

## EVIDENCES OF VALUE FOR STRENGTHENING OF UNIVERSAL IMMUNIZATION PROGRAM

**Figure 3.**
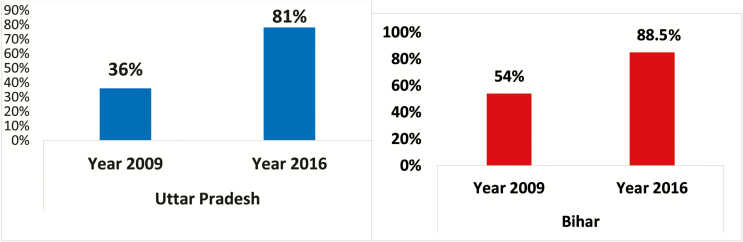
Full immunization coverage in SMNet areas. Source: UNICEF [18, 20].

Various studies have documented the SMNet’s contribution toward strengthening supplementary and routine immunization activities [17]. With the support of the SMNet, refusals in both Uttar Pradesh and Bihar have fallen to and remain at <1%, and oral polio vaccine coverage in SMNet areas is consistently >99%[18]. Evidence suggests that the SMNet’s expanded work plan to increase focus on routine immunization has led to system strengthening for communication and social mobilization activities for the Universal Immunization Program and contributed to a comprehensive increase in full routine immunization coverage in the high-risk areas: from 36% in 2009 to 81% in 2016 in Uttar Pradesh and from 54% in 2009 to 88.5% in 2016 Bihar (Figure 3) [18]. SMNet areas have also witnessed an increase in the number of children vaccinated per session site [18]. An independent evaluation showed SMNet was effective in achieving its goals of increasing immunization and building community trust in vaccination. A value-for-money analysis undertaken in 2014 revealed that SMNet has used funds in an economical manner and it is optimally utilized as per need of the polio eradication programme. The outputs of SMNet in terms of coverage and unit costs indicate a cost-efficient and economical program. Forecasted costs of SMNet for the next decade support continuing eradication interventions as the most cost-effective option [19].

## CONCLUSION AND WAY FORWARD

**Figure 4.**
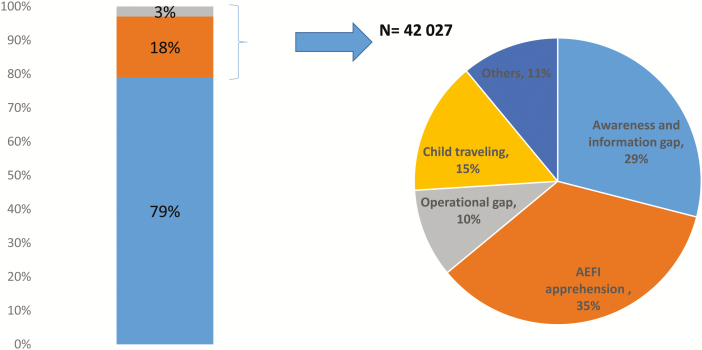
Reasons for partial immunization and no immunization of children in India, January–June 2016. Number of children aged 12–23 months monitored = 217 073. Abbreviation: AEFI, adverse event following immunization. Source: Concurrent routine immunization monitoring data. Reprinted from Feachem [24].

A large proportion of inequities in routine immunization coverage and health delivery can be attributed to inequalities in social determinants [21]. Evidence shows that addressing social determinants at the individual and community level requires effective social and community mobilization efforts [22]. Routine immunization concurrent monitoring data from 2016 shows that a major proportion (about 64%) of children missed routine immunizations because of insufficient information or understanding and could be reached through community engagement strategies used by SMNet (Figure 4) [23]. SMNet’s strategies and promotional activities demonstrated that understanding and tackling underlying social determinants contribute to sustained gains in improving immunization coverage [24].

SMNet, which evolved as a program communication network as part of the polio eradication strategy, developed and displayed some key strengths that can be used for other programs that depend on community orientation for better uptake of services, especially in underserved and marginalized communities. The network developed and displayed key strengths in terms of strong outreach and advocacy, highly skilled interpersonal communication both for individuals and groups, effective partnership leveraging, media management, and evidence-based planning. In addition, the program offers lessons in terms of robust program management systems, including communication planning, capacity building, and monitoring and supportive supervision systems.

These key lessons learned and human resources of the SMNet have the potential to be used for other government health goals and priorities, such as Reproductive, Maternal, Newborn, Child, and Adolescent Health (RMNCH+A) activities and the Global Action Plan for the Prevention and Control of Pneumonia and Diarrhea, especially in high-risk and hard-to-reach areas [25–27]. (The RMNCH+A approach was launched in India in 2013, and it essentially looks to address the major causes of mortality among women and children, as well as the delays in accessing and utilizing health care and services. The RMNCH+A strategic approach has been developed to provide an understanding of continuum of care to ensure equal focus on various life stages.) SMNet can also help increase emphasis on health promotion intervention strategies for RMNCH+A and in generating demand for health services in these hard-to-reach pockets. The gender empowerment resulting from the engagement of women in these underserved communities in the cadre of frontline workers merits further study [28, 29].

In sum, the strategies of the SMNet are replicable, proven approaches for health promotion and health-system strengthening, especially for the Universal Immunization Program, and to ensure the equity of health delivery in underserved or difficult-to-access areas. The SMNet itself remains a valuable human resource—well trained and accepted by the community—that can immediately be used for other child health initiatives and, in particular, for “reaching the unreached” with the Universal Immunization Program or broader health initiatives.

## Notes

***Supplement sponsorship.*** This work is part of a supplement coordinated by the Task Force for Global Health with funding provided by The Bill and Melinda Gates Foundation and the Centers for Disease Control and Prevention.

***Potential conflicts of interest.*** All authors: No reported conflicts. All authors have submitted the ICMJE Form for Disclosure of Potential Conflicts of Interest. Conflicts that the editors consider relevant to the content of the manuscript have been disclosed.
